# Go East for Better Honey Bee Health: *Apis cerana* Is Faster at Hygienic Behavior than *A*. *mellifera*

**DOI:** 10.1371/journal.pone.0162647

**Published:** 2016-09-08

**Authors:** Zheguang Lin, Paul Page, Li Li, Yao Qin, Yingying Zhang, Fuliang Hu, Peter Neumann, Huoqing Zheng, Vincent Dietemann

**Affiliations:** 1 College of Animal Sciences, Zhejiang University, Hangzhou, China; 2 Agroscope, Swiss Bee Research Center, Bern, Switzerland; 3 Institute of Bee Health, Vetsuisse Faculty, University of Bern, Bern, Switzerland; 4 Bee Protection Laboratory, Department of Biology, Faculty of Science, Chiang Mai University, Chiang Mai, Thailand; 5 Social Insect Research Group, Department of Zoology and Entomology, University of Pretoria, Pretoria, South Africa; University of North Carolina at Greensboro, UNITED STATES

## Abstract

The poor health status of the Western honey bee, *Apis mellifera*, compared to its Eastern counterpart, *Apis cerana*, is remarkable. This has been attributed to lower pathogen prevalence in *A*. *cerana* colonies and to their ability to survive infestations with the ectoparasitic mite, *Varroa destructor*. These properties have been linked to an enhanced removal of dead or unhealthy immature bees by adult workers in this species. Although such hygienic behavior is known to contribute to honey bee colony health, comparative data of *A*. *mellifera* and *A*. *cerana* in performing this task are scarce. Here, we compare for the first time the removal of freeze-killed brood in one population of each species and over two seasons in China. Our results show that *A*. *cerana* was significantly faster than *A*. *mellifera* at both opening cell caps and removing freeze-killed brood. The fast detection and removal of diseased brood is likely to limit the proliferation of pathogenic agents. Given our results can be generalized to the species level, a rapid hygienic response could contribute to the better health of *A*. *cerana*. Promoting the fast detection and removal of worker brood through adapted breeding programs could further improve the social immunity of *A*. *mellifera* colonies and contribute to a better health status of the Western honey bee worldwide.

## Introduction

Due to major and widespread losses of colonies [1], the health status of *A*. *mellifera*, the most widespread managed pollinator of crops and wild flora [2–3] has triggered much attention in the beekeeping as well as in the scientific communities [1,4]. Meanwhile, its Eastern counterpart, *A*. *cerana*, harbors far fewer parasites and pathogens [5–12], suggesting that it benefits from an overall higher resilience to biotic threats, and this despite its exposure to heterospecific pathogens (e.g. deformed wing virus) [12–13].

The reasons for this striking difference have been attributed to the collective defense against parasites and pathogens, which is universally expressed in eusocial insects [14–15] and more strongly so in *A*. *cerana* compared to *A*. *mellifera* [16–18]. Hygienic behavior constitutes such a defense system and reinforces the social immunity of a honey bee colony [19]. When performing hygienic brood removal, adult bees detect the cells in which diseased or dead brood occur, uncap their wax seal and extirpate the affected individuals together with the pathogenic agents from the cells and the nest, thus preventing further spread of the disease within the colony. Hygienic removal can target brood infested with *V*. *destructor* mites [16,20–23] or infected by bacteria (*Paenibacillus larvae*) [24–25], fungi (*Ascosphaera apis*) [26–28] or viruses (e.g. deformed wing virus) [29–30]. However, most of this knowledge is derived from *A*. *mellifera* and comparative data with *A*. *cerana* are scarce [20].

Hygienic behavior is a heritable genetic trait, which is commonly taken into account in *A*. *mellifera* breeding programs in order to improve the vitality of the stocks [31–32]. Such programs have been running for several years and the hygienic abilities and disease resistance of breeding *A*. *mellifera* colonies have largely been strengthened [33–34]. The assay consisting in monitoring the removal of freeze-killed brood from a comb section [35] has been acknowledged as the most conservative and reliable screening procedure to quantify the hygienic behavior of a colony [36]. We here use this method to provide the first comparison of the hygienic ability of *A*. *mellifera* and *A*. *cerana* over 72 hours. Given the better general health status of *A*. *cerana*, we hypothesized that *A*. *cerana* has a faster and higher hygienic response towards diseased brood than *A*. *mellifera*. Colonies of both species kept at the same location in Southeast China were tested simultaneously in spring and in autumn to take potential environmental and seasonal variations of hygienic behavior into account [37–38]. The results of these experiments yielded a comparative overview of the differences in freeze-killed brood removal ability between Western and Eastern honey bee colonies. We discuss whether their respective hygienic performances can contribute to the differences in health status reported in these two honey bee species.

## Materials and Methods

### Experimental colonies

We used four *A*. *mellifera* and four *A*. *cerana* full-sized colonies kept in Langstroth hives. The *A*. *mellifera* colonies were derived from unselected and heterogeneous stocks of European origin imported to China more than a century ago [39]. The *A*. *cerana* colonies were of indigenous origin (*A*. *c*. *cerana*). Since *A*. *mellifera* colonies are more populous and their workers larger in size, the hives contained 6–8 frames, whereas the smaller *A*. *cerana* colonies occupied 3–4 frames (~12,000–16,000 workers in *A*. *mellifera* colonies [40] and ~9,000–12,000 in *A*. *cerana* colonies). Given the intrusive nature of repeated evaluations of hygienic behavior, we also compared the hygienic abilities of colonies installed in observation hives. These allow the measure of hygienic removal at a higher temporal resolution without interfering with the behavior of workers. For these undisturbed measurements, five additional colonies of each species were installed in four-frame observation hives (two levels of two contiguous frames). One week before the experiment, four frames fully covered with workers (~8,000–9,000 in *A*. *mellifera* colonies [40] and ~9,000–12,000 in *A*. *cerana* colonies) and the queen were collected from each colony and placed into an observation hive. All of the colonies were located in a single apiary on Zhejiang University campus (Hangzhou, China). During the experiment, colonies were healthy and had ample honey and pollen stores.

### Freeze-killed brood assays

The hygienic ability of each colony was assessed with the standard freeze-killed brood method [35], which requires freezing capped worker brood to death with liquid nitrogen (N_2_) and monitoring their removal by workers. In order to freeze-kill the brood in each colony, a circular comb section with a high number of purple-eyed pupae was delimited with a tapered polyvinyl chloride plumbing tube (Ø = 75 mm, length = 100 mm) by twisting it down into the comb until it reached the midrib. We then poured 300 ml of liquid N_2_ into the tube—first slowly to 5 mm in height and more rapidly once the cells had started to freeze. To avoid damage, the tube was left to thaw for 20 min before it was gently removed from the comb. Finally, the treated frames were returned into their original colonies.

### Data recording and statistical analyses

In the experiment with full-sized colonies in Langstroth hives, the status of frozen cells was recorded twice on the first day and three times a day at three-hour intervals on the following days. For each recording event, the combs were taken out of their hives and the workers on their surface were gently brushed away. The areas of frozen brood were photographed before returning the comb into its original hive. The status of each cell (capped, uncapped or brood removed) was later determined based on the photographs. The experiment was performed once in late spring and repeated in mid-autumn 2013. In the experiment with observation hives, the frames were left in the hives and the status of each frozen brood cell (capped or uncapped) was recorded through the glass sides at two-hour intervals. This was done three times on the first day and four times a day during the subsequent days. These ten colonies were observed during the same period as the eight full-sized colonies in autumn 2013. Within the frozen areas, an occupied cell that was partially or completely uncapped, irrespective of the presence of brood, was considered as targeted by hygienic behavior. In both hive types, counts were performed until all of the frozen brood cells were uncapped, or terminated after three days if a colony failed to uncap all brood.

Each frozen cell was treated as an individual in a survival analysis. Kaplan-Meier plots and log-rank (Mantel-Cox) tests were used to describe and compare the hygienic abilities between species and seasons over time. We assessed the difference in hygienic task intensity experimentally induced in both species by statistically comparing the number of capped cells in the frozen area with a Student’s t-test for independent samples. The same test was used to compare arcsine-transformed percentage of brood uncapped at the first time-check and at 24 hours and thus assess the speed of hygienic detection of dead brood in these species. All statistical analyses were performed using the program SPSS (version 20.0).

## Results

Frozen comb sections contained 122 ± 12.0 and 118 ± 20.8 capped cells in *A*. *mellifera* and *A*. *cerana*, respectively. The number of freeze-killed cells showed no significant difference between the two honey bee species (Student’s t-test, *p* = 0.60).

**Fig 1:**
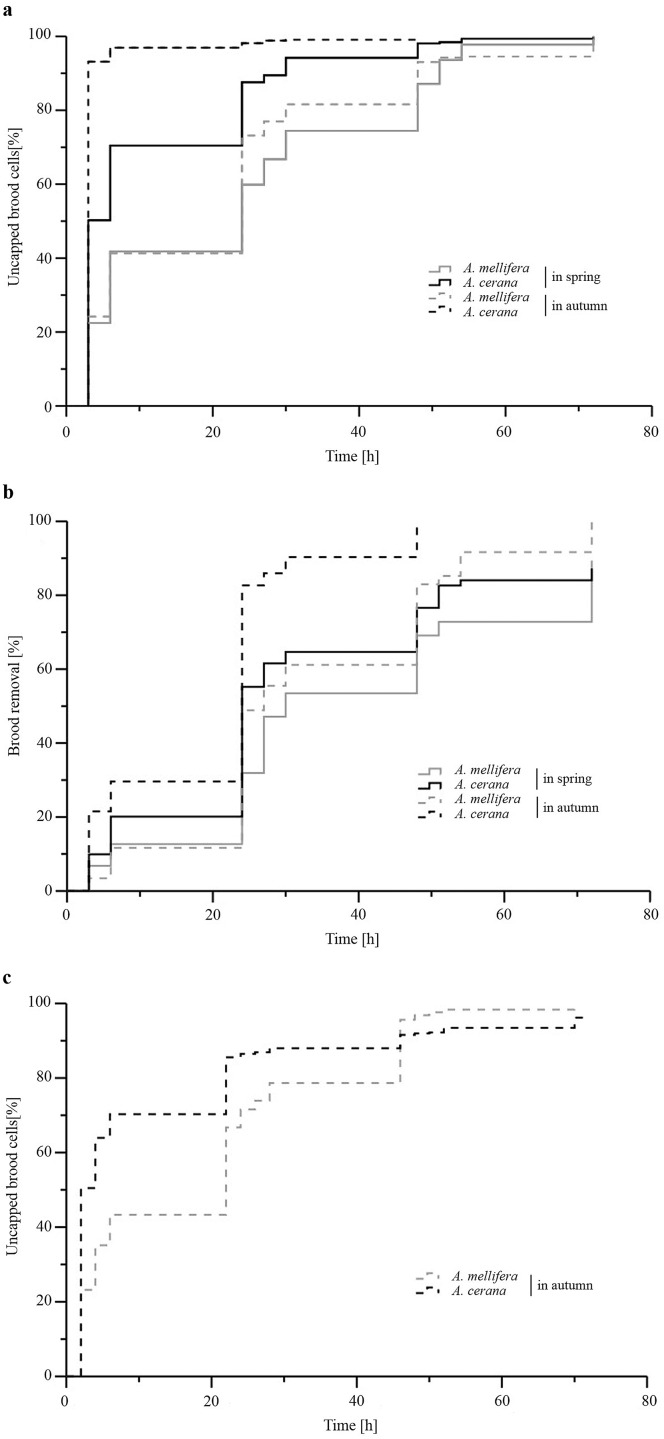
Uncapping of freeze-killed brood cells and brood removal over time in colonies of *Apis mellifera* and *A*. *cerana*. Kaplan-Meier plots are shown for cell uncapping (a) and brood removal (b) in Langstroth hives, as well as for cell uncapping in observation hives (c). For consistency with the text, we express the percentage as an increase in brood targeted by hygienic behavior instead of displaying brood survival based on the decreasing percentage of cells remaining capped. The percentage of brood uncapped or removed at a particular observation time is indicated by the value reached by the vertical line at this time and the horizontal line to the next observation.

**Fig 2:**
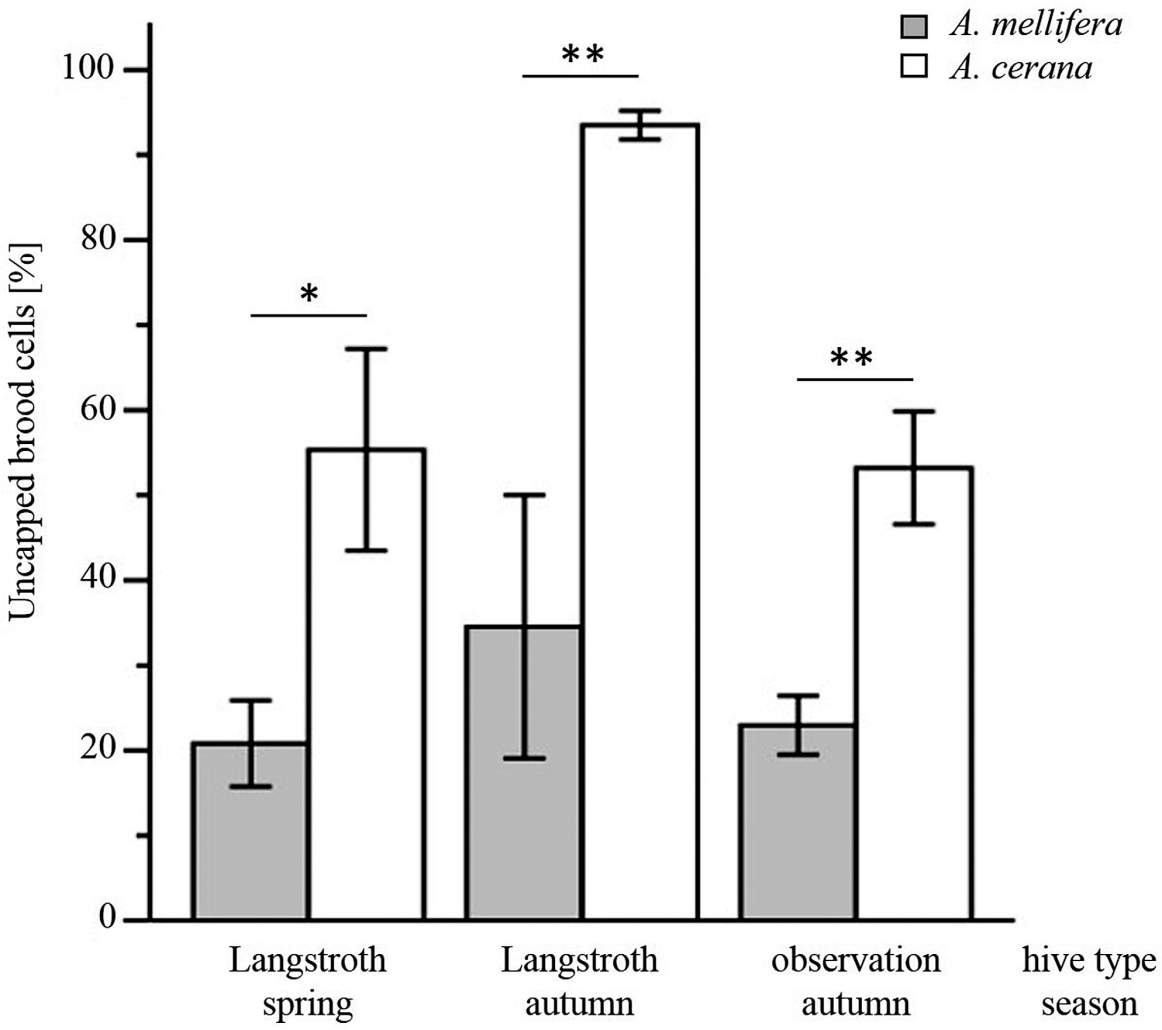
Uncapping of freeze-killed brood cells in colonies of *A*. *mellifera* and *A*. *cerana* three hours (Langstroth hives) and two hours (observation hives) after freeze-killing. Measurements are reported for spring and autumn. Means, standard errors and results of the independent samples t-test are shown (*: *p* < 0.05; **: *p* < 0.01).

All full-sized *A*. *mellifera* and *A*. *cerana* colonies uncapped all of the freeze-killed brood within 72 h. However, the log-rank test showed that both cell uncapping and brood removal of *A*. *cerana* workers was significantly higher than that of *A*. *mellifera* in spring (uncapping: χ^2^ = 752.65, *p* < 0.001, Fig 1a; removal: χ^2^ = 116.40, *p* < 0.001, Fig 1b) and in autumn (uncapping: χ^2^ = 2005.32, *p* < 0.001, Fig 1a; removal: χ^2^ = 619.54, *p* < 0.001, Fig 1b). Within the first three hours, *A*. *cerana* uncapped over twice as much dead brood as *A*. *mellifera* (Student’s t-test, *p* = 0.048 in spring, *p* = 0.006 in autumn; Fig 2). At 24 hours, *A*. *cerana* had uncapped significantly more brood (Student’s t-test, *p* = 0.010 in spring, *p* = 0.021 in autumn), but had not removed more dead pupae (Student’s t-test, *p* = 0.258 in spring, *p* = 0.287 in autumn). Both *A*. *cerana* and *A*. *mellifera*, showed significantly higher brood uncapping and removal in autumn than in spring (log-rank test, uncapping: *A*. *cerana*, χ^2^ = 797.22, *p* < 0.001, *A*. *mellifera*, χ^2^ = 26.90, *p* < 0.001, Fig 1a; removal: *A*. *cerana*, χ^2^ = 312.96, *p* < 0.001, *A*. *mellifera*, χ^2^ = 66.02, *p* < 0.001, Fig 1b).

In the observation hives, although one of the five *A*. *cerana* colonies did not complete the task by uncapping only 88.2% of the frozen brood cells over three days, this species exerted a significantly more intense hygienic behavior than *A*. *mellifera* colonies (log-rank test, χ^2^ = 641.20, *p* < 0.001; Fig 1c). *A*. *cerana* also uncapped significantly more freeze-killed brood cells during the first two hours compared to *A*. *mellifera* (Student’s t-test, *p* = 0.004; Fig 2).

## Discussion

The data clearly show that Eastern honey bees, *A*. *cerana*, are consistently faster than Western honey bees, *A*. *mellifera* and thus probably more efficient in hygienic brood detection (as shown by their uncapping of cells containing dead brood) and removal. This higher efficiency was irrespective of season and of hive type and was expressed within the first few hours of exposure to freeze-killed brood. Both species performed hygienic behavior to a higher degree in autumn compared to spring.

In this study, we monitored hygienic behavior in a single population of introduced *A*. *mellifera* and of endemic *A*. *cerana*. *A*. *mellifera* was imported in China around 1896 [39], well before breeding for hygienic behavior started in the North America or in Europe [41] and selection for hygienic behavior has not been practiced since this time in the sampled population. The domesticated *A*. *cerana* population used in our experiments originated from indigenous wild *A*. *cerana* Fabricius colonies of the mainland population. For both species, the queens have been naturally and locally mated in the populations sampled. This indicates that our sample is representative for non-bred Chinese *A*. *cerana* and *A*. *mellifera* populations of the southeast region of mainland China. In line with this fact, the brood removal after 24h measured in the *A*. *mellifera* population studied of 38.9 ± 12.2% and 59.2 ± 28.2% in spring and in autumn, respectively, corresponded to that reported for colonies of intermediate hygienic abilities (5 to 65%) [36]. Hygienic behavior in honey bee colonies can be highly variable among and within populations and subspecies, due to differences in habitat, genetic lineage and geography [23,33,38,42]. Repeating our experiment in other regions where both species co-occur is thus necessary to generalize the results.

When comparing freeze-killed brood hygiene between the two experimental seasons, we found that the colonies of both honey bee species detected and removed dead brood faster in autumn than they did in spring (Fig 1a). Autumn is the season at which *A*. *mellifera* colonies are at the greatest risk of viral infections in Europe [43]. Indeed, it is during this season that the prevalence of deformed wing virus and acute bee paralysis virus, two harmful viruses closely associated with *V*. *destructor* infestation, is peaking in *A*. *mellifera* [44]. Severe mite infestations, which are very likely to promote virus infections [45], are also observed in autumn in China [46]. Similarly, sacbrood virus, the most common pathogen of *A*. *cerana*, is more prevalent in autumn and late winter [46]. Hence, hygienic behavior could be reinforced in both honey bee species by means of natural selection, when pathogenic pressures are at their highest.

*A*. *cerana* colonies detected and removed dead brood significantly faster compared to *A*. *mellifera* (Fig 1), indicating a more efficient hygienic behavior. This advantage remained independently of hive type and season, suggesting that it is an intrinsic characteristic of the Eastern honey bee. Larger colonies might be more efficient in hygienic behavior, because more workers can be allocated to this task. However, it is the less populous *A*. *cerana* that showed faster hygienic behavior, suggesting that colony size is less relevant in this regard. A fast hygienic response is likely to have an adaptive value since a rapid brood removal should limit the transmission of fast replicating pathogens (e.g. viruses and bacteria) within the colony [47–52]. The sooner diseased brood is removed (i.e. the fewer viruses or bacteria can replicate), the lower are the chances for hygienic adult workers to become contaminated *per os* with pathogenic amounts of microorganisms [53]. At 24 hours, the difference in degree of hygienic behavior expressed by full colonies of both species decreased, suggesting that hygienic removal in the first few hours after brood damage are important for effective hygiene. Evaluating hygienic capacities of colonies used in selection programs in the first hours after brood killing might thus improve their success. Testing *A*. *mellifera* colonies highly selected for hygienic behavior and disease resistance [33–34,54] for their brood removal capacity as soon as a few hours after brood killing could also test the proposed link between rapidity of diseased brood removal and good colony health.

The trigger of hygienic behavior relies on the stimulus intensity emitted by unhealthy brood and on the olfactory sensitivity of workers [52,55–57]. Thus, workers that initiate the hygienic behavior by detecting and uncapping the cells show a greater olfactory sensitivity compared to those that complete the process by removing the brood [57]. The difference in efficiency observed between *A*. *cerana* and *A*. *mellifera* may result from the superior olfactory sensitivity reported for *A*. *cerana* workers [58–59]. Alternatively, but not mutually exclusive, a higher susceptibility of brood to antagonists in *A*. *cerana* [60] might contribute to this difference by producing signals leading to their removal earlier and in greater quantity. Further studies are required to identify the proximate mechanisms underlying the faster hygienic response of *A*. *cerana* and the possible contribution of this trait to the better health of honey bees. Confirming this link would provide an opportunity for further improving the success of existing breeding programs for hygienic behavior by integrating fast detection and removal of diseased brood ahead of the 24 or 48 h currently used as standards.
